# Network between Cytokines, Cortisol and Occupational Stress in Gas and Oilfield Workers

**DOI:** 10.3390/ijms21031118

**Published:** 2020-02-07

**Authors:** Marcella Reale, Erica Costantini, Chiara D’Angelo, Luca Coppeta, Rocco Mangifesta, Srinivas Jagarlapoodi, Marta Di Nicola, Luca Di Giampaolo

**Affiliations:** 1 Department of Medical, Oral and Biotechnological Sciences, University “G. d’Annunzio” Chieti-Pescara, 66100 Chieti, Italy; erica.costantini@unich.it (E.C.); chiara.dangelo@unich.it (C.D.); rocco.mangifesta@unich.it (R.M.); srinivas.jagarlapoodi@unich.it (S.J.); marta.dinicola@unich.it (M.D.N.); luca.digiampaolo@unich.it (L.D.G.); 2 Department of Occupational Medicine, Occupational Health Service, University of Rome Tor Vergata, Viale Oxford 81, 00133 Rome, Italy; Lcoppeta@gmail.com

**Keywords:** cytokines, cortisol, decision latitude (DL), job stress, oil and gas field worker, psychological demands

## Abstract

To test whether gas and oil field work is accompanied by stress and altered immune function, the perception of workplace stress, levels of salivary cortisol, plasma levels, and mononuclear cell production of cytokines were examined in 80 healthy workers recruited among a population of operators on gas and oilfields. Specific questionnaires for determining the perception of anxiety, occupational stress, and subjective symptoms were administered. Salivary cortisol and cytokines plasma levels were evaluated by Elisa and to investigate immune function, both spontaneous and PHA- or LPS-induced expression and production of cytokines were assessed by qRT-PCR. Workers showed medium stress levels at work, with growth and increased motivation for work, and based on salivary cortisol concentrations, were divided into two groups of ≤10 ng/mL (*n* = 31) or >10 ng/mL (*n* = 49). Statistically significant higher plasma levels of IL-6, while lower TNFα, were detected in workers with cortisol >10 ng/mL. Also, BMI, DL, JD and Job strain were significantly higher in workers with cortisol >10 ng/mL. Thus, even modest variations of cortisol might have a role in the modulation of immune response and worker’s vulnerability to health imbalance.Thus, the evaluation of immune status, in addition to cortisol levels, could be useful to prevent illnesses; exacerbation of pre-existing conditions; morbidity; and consequent absences from work, with economic repercussions.

## 1. Introduction

In the last decades, research has pointed out the role of stress in the variation of the physiological cross-talk between the brain, immune, and endocrine systems. Brain-immune cross-talk is greatly influenced by mental states and psychosocial factors. Communication between the Central Nervous System (CNS) and the immune system is essential to establish homeostasis and cytokines; hormones and cell-to-cell interactions allow for the integration and reciprocal regulation of the CNS and immune system.

Cytokines are the main intercellular protein messengers that are secreted by many cell types and can act locally on the cell of origin itself or distantly and each cytokine can modulate more than one biological function, so that the complex interactions between the cytokines form the “cytokine network”, and the overall effect will depend on the type of cytokines, the receptors involved and the context of the response. The synthesis and release of cytokines, which are an important part of the immune response, are generally protective but can be dangerous if inappropriate or excessive. Based on cytokine production, the CD4 T-cells can be subdivided into Th1, Th2, Th17, and Treg subsets. Th1 cells release proinflammatory cytokines such as interferon (IFN) γ, Interleukin (IL)-2, tumor necrosis factor (TNF) α, IL-6, IL-8, and IL-1β that drive inflammation and cellular-mediated responses, and Th2 cells produced cytokines such as IL-1ra, IL-4, IL-5, and IL-10 that are associated with humoral immunity and anti-inflammatory properties [1]. Th17 cells, secreting IL-17, IL-21 and IL-22, promote the development of inflammatory responses, while Treg cells that secrete TGF-β and IL-10 were related to Th17 cells, and antagonize each other. The balance of Th1/Th2 and Treg/Th17 is the key in maintaining immune homeostasis. Also, macrophages, B lymphocytes, dendritic cells, mast cells, endothelial cells, and fibroblasts are very important cytokine-producing cells, and all produce detectable amounts of IL-1, IL-6, IL-8, IL-12, IFNβ, and TNF [2].

Cytokines, in addition to the regulation of immune processes, play a key role in the communication between the immune system and the rest of the body. Cells, throughout the body and CNS, produce and release cytokines that also coordinate and regulate the communication of the neuroendocrine-immune network. The peripheral immune system can affect the cytokine balance in the brain, thereby altering mood and behavior [3,4], and can enhance the activity of the Hypothalamic–Pituitary–Adrenal (HPA) neuroendocrine branch of the stress response [5]. High levels of inflammatory cytokines stimulate the HPA axis to release cortisol that inhibits inflammatory cytokine production [6]. Dysregulation of the HPA axis results in the lack of suppression of the immune system, known as glucocorticoid resistance [7].

The HPA axis is a key component of the neuroendocrine system that controls stress responses to the environment, and dysregulation of neuroendocrine, cardiovascular and metabolic systems, but increased incidence of viral infections may be due also to job stress. Work stress has increasingly become an important public health problem. Several studies have pointed out the association between job stress and immune parameters evaluated in blood and other biological fluids [8,9,10,11].

Many studies have been conducted to highlight the relationship between stress at work and physical and mental health of workers in the gas and oil industry, showing that working conditions such as shifts, work requirements, and the working environment influence the different perception and difficulties of work and that the working capacities decreased with age, working time and stress levels [12,13]. Several studies have reported higher levels of nervousness, mental fatigue, anxiety, sleep problems, and overweight in gas and oil extraction workers [14,15]. On the other hand, several studies have underlined that oil and gas extraction workers do not present mental health problems [16,17]. Although it is known that the offshore work presents unique situations and workers are more frequently exposed to stressful conditions, also for oil and gas onshore workers, the lack of spatial separation between the onshore workplace and the onshore residence, work-load and repetitive work, unbalanced and poor quality foods represent important stressors, and consequently, the work has been described as difficult, stressful and potentially dangerous.

A situation perceived as stress triggers a series of mechanisms, including the hyperactivity of the HPA axis and elevated cortisol concentrations. The bidirectional relationship between cytokines and cortisol contributes to the maintenance of immune system homeostasis and is critical to regulating inflammation and maintaining health [18,19]. We aimed to evaluate the cytokines and cortisol levels in healthy onshore workers and to explore if work conditions are associated with perceptions of occupational strain and health. Besides, we will investigate the association between cortisol level and cell responsiveness to different stimuli, by evaluating cytokines production, as mediators of immune responses.

## 2. Results

### 2.1. Salivary Cortisol Levels

**Figure 1:**
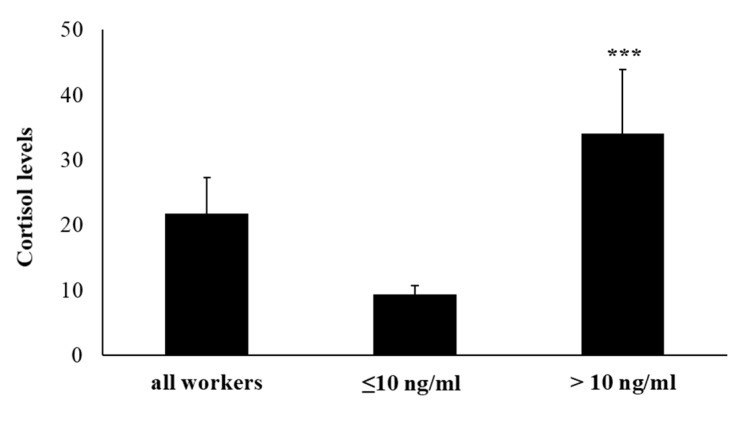
Salivary cortisol levels in all enrolled workers. Workers were divided into two groups based on salivary cortisol levels ≤ or > of 10 ng/mL. Data are reported as mean ± S.D. *** *p*-value ≤ 0.001.

The concentration of salivary cortisol has been evaluated as a marker for the activation of the HPA-axis and as a biomarker of stress. Cortisol was measured using a commercial (solid phase) ELISA Kit. As it is known, salivary cortisol shows a diurnal rhythm, reaching its highest levels early in the morning, before beginning to decline throughout the day with the lowest levels at night. In our samples, collected in the morning, at 8:00 a.m., we have detected an average value of 21.72 ng/mL for the enrolled workers. Subjects were divided into two groups based on their salivary cortisol concentrations of ≤10 ng/mL (*n* = 31; range: 5.4–10.1) or >10 ng/mL (*n* = 49; range: 10.9–41.3). In 61.25% of the recruited workers, salivary cortisol was more than 10 ng/mL. Data on salivary cortisol analysis are reported in Figure 1.

### 2.2. Perception of Anxiety, Job Stress, and Physical Symptoms

In our study sample, employees were aged between 27 and 47 years. Nine workers were coordinators, 12 workers were in supervision positions, and the others were in the quality of the operator. Enrolled workers reported satisfactory mental and physical health. However, two workers reported perception of stress at work, fatigue, difficulties in detaching from work, and sleeping problems, all with moderate extents. Most of the workers stated that they did not expect any long-term adverse health effects as a consequence of spending time alternatively at work in onshore oil and gas installations or at home. About physical symptoms and complaints, only 17 workers reported having pain, in detail, six suffered from a stomach ache accompanied by diarrhea, lasting no more than 2 days, five abdominal pain, four headaches and dizziness, and two had a cold. Table 1 shows the demographic data and results of the questionnaire administered to all enrolled subjects, to determine their perception of anxiety through the state-trait-anxiety inventory (STAI), and job stress, considering job demand (JD), decision latitude (DL), social support (SS), job insecurity (JI), and job strain. Overall, workers had medium stress levels at work, with increased motivation for work. Dividing workers according to cortisol levels, it is possible to see how, in subjects with a higher cortisol level, the tests show slightly increased values. DL and JD showed a statistically significant difference between the group with salivary cortisol ≤10 and group with cortisol >10 ng/mL (*p* < 0.05 and *p* < 0.001, respectively). Workers with cortisol >10 ng/mL showed significantly higher weight and body max index (BMI) (*p* ≤ 0.001) than workers with cortisol ≤10. All workers were long-term contractors with more than 7 years’ experience in the extraction installation. Workers with levels of cortisol >10 ng/mL are those with the most years of work at extraction installation.

### 2.3. Hematological Blood Parameters

In this study, the evaluation of blood cell populations number was determined. No, significant differences in overall blood cells number were detected between the group with cortisol ≤10 and group with cortisol >10, as reported in Table 2.

### 2.4. Plasma Cytokine Profile

**Figure 2:**
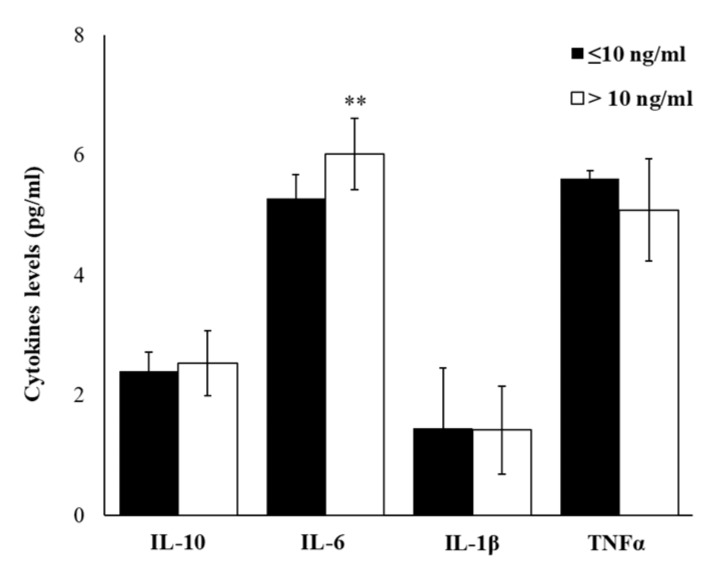
Plasma levels of pro- and anti-inflammatory cytokines. Plasma levels of IL-10, IL-6, IL-1β, and TNFα in enrolled workers, according to salivary cortisol levels ≤ or > of 10ng/mL. Data are reported as mean ± S.D. ** *p*-value ≤ 0.01.

In our enrolled 80 subjects, matched for age and BMI and without disease conditions, injury or medication, we have evaluated plasma levels of several Th1 and Th2 cytokines. Based on cortisol levels ≤10 ng/mL or >10 ng/mL, the results obtained from cytokine detections were reported. Figure 2 showed that in workers with cortisol >10 ng/mL, plasma levels of IL-6 are statistically significantly higher (*p* ≤ 0.01), while no different levels of IL-10, IL-1β and TNFα were observed between two groups.

### 2.5. Cytokines Gene-Expression

**Figure 3:**
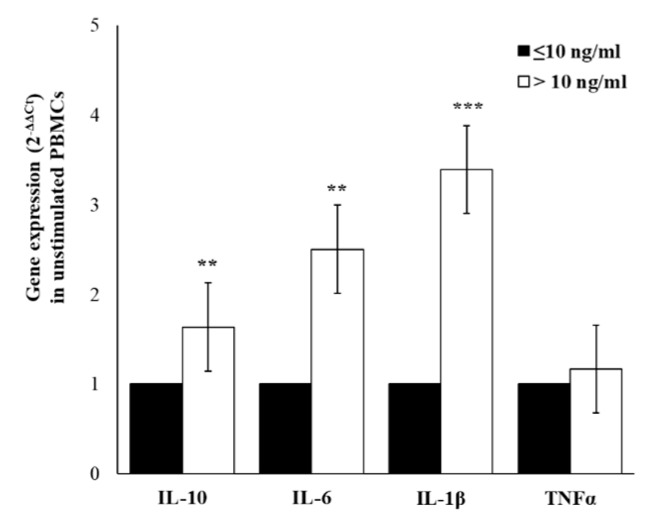
Cytokines gene expression. Basal IL-10, IL-6, IL-1β, and TNFα expression levels in PBMCs from enrolled workers with salivary cortisol levels ≤ or > of 10 ng/mL. Data are reported as mean. 2^−ΔΔCt^ ± SE. ** *p*-value ≤ 0.01; *** *p*-value ≤ 0.001.

**Figure 4:**
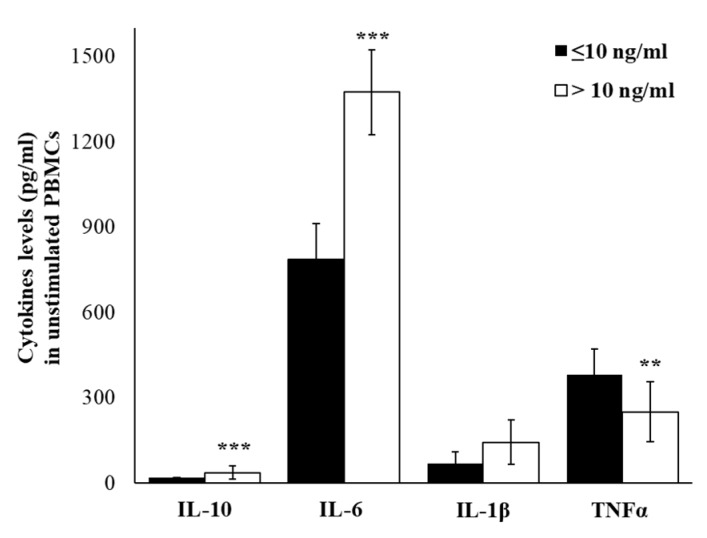
Basal cytokine production. IL-10, IL-6, IL-1β, and TNFα levels in supernatants of untreated PBMCs of enrolled workers with salivary cortisol levels ≤ or > of 10 ng/mL. Data are reported as mean ± S.D. ** *p*-value ≤ 0.01; *** *p*-value ≤ 0.001.

Since plasma cytokine levels represent the sum from all sources, it is uncertain whether the differences between groups were due to altered secretion by immune cells, non-immune cells, or both sources, or were due to rate of clearance. To investigate if differences in plasma levels of cytokine mirror a different gene expression profile in cell-producing cytokines, we have evaluated the cytokines expression in peripheral blood mononuclear cells (PBMC) isolated from enrolled workers. Figure 3 shows the differences between the two groups of workers based on cortisol levels, with higher spontaneous expression levels of all cytokines in PBMCs from workers with cortisol >10 ng/mL. The higher levels were detected for IL-1β (about 3.4 times respect to group with cortisol ≤10) and for IL-6 (about 2.4 times respect to group with cortisol ≤10). In accord with expression data, also the levels of released cytokines, detected in cell-free supernatants, were higher in the group with cortisol >10 ng/mL. The significantly higher levels of IL-6 (*p* ≤ 0.01) and IL-10 (*p* ≤ 0.01), and lower levels of TNFα (*p* ≤ 0.01) were observed in cortisol >10 groups. The increasing trend, although in smaller and not significant amounts, was observed for IL-1 β (Figure 4).

**Figure 5:**
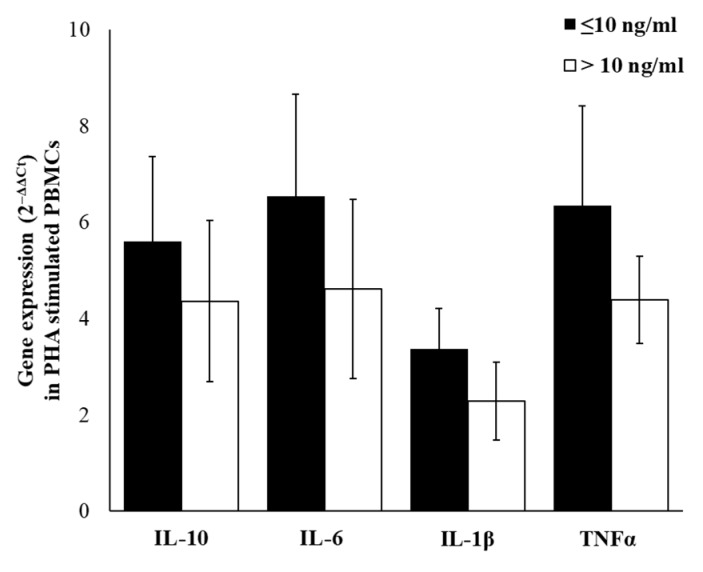
PHA-induced cytokine expression. IL-10, IL-6, IL-1β, and TNFα gene expression in PHA-stimulated PBMCs of enrolled workers with salivary cortisol levels ≤ or > of 10 ng/mL. Data are reported as mean 2^−ΔΔCt^ ± SE.

Due to the low amount of cytokines produced by resting immune cells and to consider differences related to complex cell–cell interactions, we have stimulated PBMCs with the mitogenic lectin, Phytohaemagglutinin (PHA). In PHA-stimulated PBMC, we observed an up-regulation of all selected cytokines and, even if not statistically significant, the up-regulation of IL-6, TNFα, IL-10, and IL-1β respectively, is most evident in a group with cortisol ≤10 ng/mL for (Figure 5).

**Figure 6:**
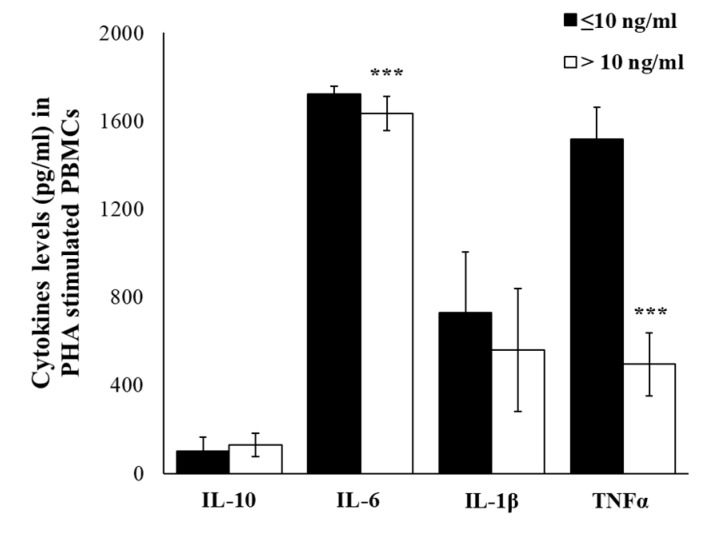
PHA-induced cytokine production. IL-10, IL-6, IL-1β, and TNFα levels in supernatants of PHA-treated PBMCs of enrolled workers with salivary cortisol levels ≤ or > of 10 ng/mL. Data are reported as mean ± S.D. *** *p*-value ≤ 0.001.

To evaluate the relation between gene expression and production, we have analyzed levels of IL-10, IL-6, IL-1β, and TNFα in the supernatants of PBMCs incubated with PHA. The relevant result was that in the supernatants of PHA-treated cells, the levels of released cytokines were in accord with gene expression levels, with significantly higher levels of IL-6 (*p* ≤ 0.001) and TNFα (*p* ≤ 0.001) in the group with cortisol ≤10 ng/mL. No significant differences were detected, for IL-1β and IL-10 levels, between groups (Figure 6).

**Figure 7:**
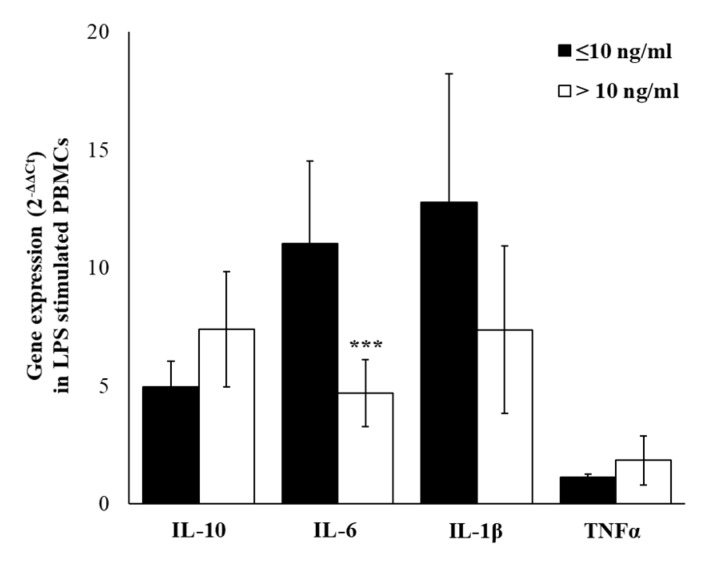
LPS-induced cytokine expression. IL-10, IL-6, IL-1β, and TNFα levels in LPS-stimulated PBMCs of enrolled workers according to salivary cortisol levels ≤ or > of 10 ng/mL. Data are reported as mean 2^−ΔΔCt^ ± SE. *** *p*-value ≤ 0.001.

**Figure 8:**
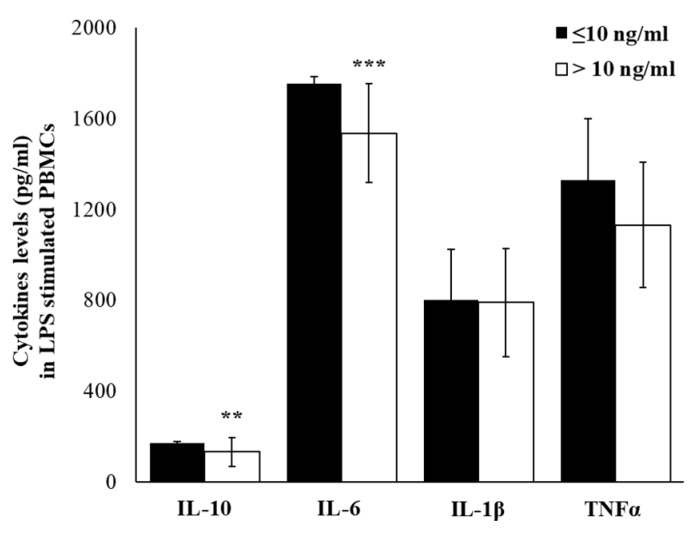
LPS-induced cytokine production. Levels of cytokines detected in the supernatants of LPS-stimulated PBMC from workers with cortisol ≤10 ng/mL or cortisol >10 ng/mL. Data are reported as mean ± S.D. ** *p*-value ≤ 0.01; *** *p*-value ≤ 0.001.

The lipopolysaccharide (LPS), a component of the outer wall of Gram-negative bacteria and a well know inductor of inflammatory cytokines production, was used to assess if cortisol levels may influence the immune activation and inflammation. Thus, we have treated PBMCs of our workers, and although results showed that LPS induced an increase of cytokines expression, in workers with high cortisol levels a significantly lower IL-6 level (*p* ≤ 0.001) was detected. Although not statistically significant, in LPS-stimulated PBMCs, we have detected lower IL-1β and higher IL-10 and TNFα gene expression levels (Figure 7). In the supernatants of LPS-stimulated PBMCs, IL-6, IL-1β, and TNFα were the most abundantly produced cytokines in both cortisol ≤10 and cortisol >10 groups. Significant lower levels of IL-6 (*p* ≤ 0.001) and IL-10 (*p* ≤ 0.01), and although not statistically significant, lower levels of TNFα and IL-1β, were detected in the group with cortisol >10 (Figure 8). Based on these results, we have hypothesized that the responsiveness to inflammatory stimulus in workers with higher salivary cortisol levels was reduced with respect to the responsiveness of PBMC from workers with lower salivary cortisol levels.

## 3. Discussion

Workplace or the working environment could represent a specific source of stress for workers, determining an increased risk of disease development and discomfort in private and social life. Workplace stress has increased significantly over the last three decades representing a big problem for employees and organizations. High levels of work stress also involve an increase in absenteeism and turnover, chronic exhaustion or other negative long-term health conditions and consequently with higher costs for organizations and society. Stress can be related to the systemic inflammation with elevated peripheral cytokine levels, negatively affecting the health [11]. In fact, elevated systemic levels of cytokine may be responsible for HPA axis activation leading cortisol secretion, which by inhibition of the nuclear transcription factor-κB (NF-κB) signaling, may dampen the inflammatory response [20]. Thus, cortisol could represent a link between stress and inflammation, and levels of both cortisol and cytokines could be strong indicators of the imbalance between the immune and endocrine systems in stressful conditions [21]. Several studies have evidenced that activation of a bidirectional feedback loop between the cytokines and cortisol play a key role in the appropriate function of the HPA axis, maintaining homeostasis of the immune system [22,23,24].

Cortisol is mainly present in the blood and is transported by binding proteins. Only a small fraction is the active component and it may be found in the same amount also in saliva. In the last decade, saliva has already been used as a biological fluid for diagnosis and clinical monitoring of several diseases, for its ease of collection and conservation and presence of many biomarkers [25,26]. Salivary cortisol level, that is independent of the salivary flow rate, is a valid indicator of the plasma free cortisol concentration [27], and due to its feasibility to collect, as well as being non-invasive and without pain and/or stress for patients [28,29,30], and having low collection cost [31], the evaluation of salivary cortisol is very common in research and laboratory studies.

In this study, we have investigated the job stress, perception of anxiety, salivary cortisol, and cytokine levels in workers in the gas and oil industry. Questionnaires for determining the perception of anxiety, occupational stress, and subjective symptoms were administered to each subject, and saliva and peripheral blood have been collected to evaluate cortisol and cytokine axis. Sampling was performed in the same conditions and time in all the subjects to exclude the influence of circadian variation [32], circumstances [33], food intake [34], or intra-individual day-to-day variability [35,36].

Results of our study showed that 61.25% of enrolled workers had salivary cortisol levels higher than 10 ng/mL, and the analysis of the job content questionnaire (JCQ) and STAI questionnaires showed that in this worker’s group there are safe a medium job-stress levels with increased motivation for work: a safe level of stress at work means that a worker perceives good control over the activities to be carried out and a substantial work demand.

Nakata et al. have reported that decreased natural killer cell numbers, decreased T cell subsets and CD4+/CD8+ ratio, as well as increased inflammatory markers, are related to job stress [11]. Since differences in immune cell counts could not reflect changes in immune function, as a more valid assessment of the immune system activity, we have evaluated immune cells responsiveness comparing spontaneous or PHA- and LPS-induced cytokines expression and production as a functional assay of immune cells in workers with cortisol ≤10 ng/mL and workers with cortisol >10 ng/mL.

Cytokine production is not organ or cell-specific, but can occur everywhere in the human body; in addition, other than immune cells, also fibroblasts, endothelial and epithelial cells, neurons, microglia, and astrocytes can produce cytokines. Cytokines may be implicated in the development of autoimmune diseases [37,38,39], cardiovascular diseases [40,41,42], sleep onset and regulation [43], pain sensitivity [44,45,46], cell differentiation, and programmed cell death [47]. Peripherally produced cytokines can affect neuroendocrine and brain function, playing an important role in Alzheimer’s disease (AD) [48,49,50], cognitive dysfunction [51], clinical depression [52,53], and dementia [54]. Thus, understanding how the dysregulation of cytokines and how cytokine signaling contribute to human disease, as well as how cytokines action could be limited to avoid the pathogenic effects of their superproduction, is of paramount importance.

The present study aimed to examine the expression and production of these inflammatory cytokines and their relationship with cortisol levels in gas and oil extraction workers that spend alternatively 2 weeks at work and 3 weeks at home, during the year.

It is known that cortisol may display an immunomodulatory activity [55,56,57,58,59], causing changes in cellular migration, proliferation and cytokine secretion [60,61,62,63,64,65,66]. High cortisol levels might be related to the Th1/Th2 cytokines balance, resulting in immune dysregulation rather than immunosuppression. High cortisol levels might suppress the Th1-mediated cellular immune response, increasing the risk of infectious diseases. In the meantime, it could enhance Th2-mediated humoral immune response which can increase the risk of autoimmune and allergic diseases. Our results showed a significantly higher plasma level of IL-6, lower levels of TNFα and non-significant differences of IL-1β levels in workers with cortisol levels >10 ng/mL.

Cytokine administration increases cortisol release, and inflammatory cytokines may act through the modulation of the cortisol receptor’s (glucocorticoid receptor) expression and function. Glucocorticoid receptors are found in human immune cells such as lymphocytes, mononuclear cells, and granulocytes, and cytokines released by these cells can modulate the release of cortisol, establishing a mutual control [67,68]. To understand how cortisol levels may modulate the expression and production of cytokines of peripheral immune cells, we have isolated PBMC from workers with cortisol levels ≤ or > of 10 ng/mL.

Although we did not find a significant correlation between salivary cortisol levels and the expression of inflammatory cytokines, our results highlight that the spontaneous gene expression of cytokines was higher in PBMC from the group with salivary cortisol >10 ng/mL, suggesting that cortisol may be involved in the control of the cytokine expression in accord with the several studies that reported a relationship between stress and Th1/Th2 cytokine levels [69,70,71,72,73]. The differences between the levels of cytokines released spontaneously by the PBMCs of the two groups are less marked than the differences observed in gene expression and, surprisingly, in the group with cortisol >10 ng/mL at the higher TNFα expression corresponds a lower TNFα concentration present in the supernatants. Since the secretion of cytokines showed distinct secretory pathways (i.e., TNFα used canonical while IL-1β non-canonical secretory pathway), we argue that cortisol may negatively modulate, above all, the canonical secretory pathway of TNFα, as supported also by the same pattern of plasma cytokine levels.

Then we have used PHA to activate a cascade of signaling events, including the up-regulation of NF-κB, activator protein-1 (AP-1) and nuclear factor of activated T cell (NFAT) and to lead the amplification of the immune response by establishing a positive feedback circuit [74,75]. In our workers, the PHA-induced expression and production of cytokines were lower in the group with cortisol >10ng/mL. Exposure of PBMC to LPS, a classical inductor of inflammation, leads to lower expression IL-6 and IL-1β, higher expression of IL-10 and TNFα, but lower production of IL-6, IL-10 and TNFα in workers with salivary cortisol levels >10 ng/mL. This lower PBMC’s responsivity to PHA or LPS stimulation may be due to overload, as a result of excessive diffuse immunological over activation, making activated PBMC not able to respond to stimulation. We hypothesized a mechanism which protects cells from the excess of stimulation and contributes to the regulation of cell homeostasis.

Previous studies showed that in serum and plasma, IL-10 was increased in response to an academic examination stressor [76], and that IL-10 rose in the short-term simultaneously with pro-inflammatory markers in response to manipulated stress and pain but then decreased with protracted pain [46], which may explain why both higher and lower levels of IL-10 have been observed in individuals with pain [77]. We detected higher circulating levels of IL-10, as well as also spontaneous or LPS-induced expression in subjects with cortisol >10. TNFα, IL-1, and IL-6, the main pro-inflammatory cytokines, and IL-10, a powerful anti-inflammatory cytokine, are produced by monocytes and macrophages in response to LPS. IL-10 has been shown to inhibit the expression of these pro-inflammatory cytokines and thus limit the inflammatory response [78]. Thus, we suggest that in workers with cortisol level >10, the IL-10 levels modulating the balance between pro-and anti-inflammatory cytokines regulate also the immune response toward a pathogen.

Our results show that in our workers, 2 weeks of onshore work in oil and gas installations followed by rest periods, have not negative health effects or significant effects on stress or self-perceived psychosocial risk in the workplace (at least not with the questionnaires used in this research). Globally, the workers enrolled in this study do not have very high cortisol values, which is in accordance with the low cytokines levels and mild work stressors. There is evidence, in this study, that individual differences in cortisol levels were low and not associated with health dysfunction. This could be interpreted as a physiological adaptation to the job and its demands and as the use of individual coping resources that could be responsible for the normalization of psychological stress, as suggested by Lian et al. [79]. It could be assumed that our workers can handle the occupational stress, in fact, no significant health impairments such as headache, fatigue, and sleep quality and irritability were reported.

The immune–endocrine interactions between cytokines and cortisol, in response to the moderate work stressors, are still unclear. The interesting results of this study are that the higher cortisol levels, even non-elevated, are linked to the activity of immune cells and could be risk factors for immune dysregulation. In fact, lower expression and production of pro-inflammatory cytokines were found in LPS-stimulated PBMCs from individuals with higher cortisol levels, suggesting reduced ability to respond to bacterial infections and increased vulnerability among workers to infectious diseases, which is in accordance with previous studies showing that long-term exposure to cortisol may impair the response of the immune system by a downregulation of hormonal receptors [80]. Our results underlined a cross-talk between the production of proinflammatory cytokines and cortisol levels, which is in accordance with the hypothesis that a hypoactive HPA axis might contribute to the development of inflammatory conditions.

Several studies have highlighted the association between the increased risk of dementia and AD with work-related psychosocial stress [81]. In fact, in AD, other than the main pathological hallmarks such as Amyloid-β plaques and neurofibrillary tangles, also the increased levels of pro-inflammatory cytokines and reduction of the anti-inflammatory system are a central feature of the disease [82,83,84]. The results of our study reinforce the evidence of the link between stress, an inflammatory cytokine, and dementia risk. Chronic inflammation, caused by lifestyle factors and stress, keeps cortisol levels high, influencing the functioning of the immune system. An uncontrolled immune system that responds to persistent inflammation can lead to problems such as increased disease susceptibility, increased risk of cancer, a tendency to develop food allergies, and increased risk of autoimmune disease.

Even though it would be premature to overstate the significance of this finding, we suggested that cytokines are promising biomarkers for occupational stress research. Since health-related, anxiety and occupational stress data were all self-reported by the participants, and all participants were male, additional studies with a large sample size could clarify the relationship between cortisol and immunity in gas and oil extraction workers.

The strengths of our study include the evaluation of cortisol and cytokines levels collectively with strict attention to population characteristics, and evaluation of PHA- and LPS-induced cytokines expression to reflect the response to a different inflammatory stimulus. Limitations include the use of a self-report measure of perception of symptoms, drinking and smoking habits, and the single determination of cortisol. Thus, we enrolled workers who did not differ in their work shifts, in smoking and drinking habits, and their wake up in the morning, and hematological and blood pressure parameters or demographic variables were collected. Saliva samples were collected at the same time, and although the cortisol in the saliva is stable at room temperature for several days, we kept the sample at -80 until the assay.

## 4. Materials and Methods

### 4.1. Participants

A total of 80 healthy workers were recruited among a population of operators on gas and oil extraction. All participants fulfilled the criteria of in- and exclusion, i.e., they were in good physical and psychological health and they had no history of cardiovascular disease, allergy, asthma, diabetes, infectious disease, pain clinical psychosomatic and psychiatric diseases, elevated cholesterol, liver and renal diseases, chronic obstructive pulmonary disease, and rheumatic diseases. No drugs or medications were administered during the previous 2 weeks. The purpose and procedures of the study were explained to all potential participants, and informed consent was obtained from those who decided to participate. The work of enrolled subjects takes place by turns, with 2 weeks of work without any breaks followed by rest periods of 3 weeks at home. Thus, during the year a worker alternatively spends their time at work away from their loved ones, and at home. To evaluate job stress, measured by the job strain model (high job demands and low job control), we have administered specific questionnaires to workers. Questionnaires and saliva and blood samples were collected at work shifts after 2 weeks of work in onshore oil and gas installations.

The study, approved by the Ethics Committee of the Provinces of Chieti and Pescara and the “G. d’Annunzio” University of Chieti-Pescara (Ethics Committee Project No.15 the 03/09/2015), was performed in accordance with the ethical standards laid down in the 1964 Declaration of Helsinki.

### 4.2. Characteristics of Enrolled Subjects

Details of lifestyle (e.g., smoking and alcohol consumption), marital status, educational and occupational grade (coordinator, supervisor or operator), information on physical symptoms, and complaints (diarrhea, abdominal pain) were recorded for all participants and reported in Table 3. The frequency of drinking alcohol and cigarette smoking during work in onshore oil and gas installations, which is inevitably restricted by their work environments, was assessed in a self-completed questionnaire

### 4.3. Analysis of Anxiety, Occupational Stress, and Perception of Symptoms

Work-related outcomes related to lifestyle can favor the appearance of chronic stress in the worker. To determine the perception of anxiety, occupational stress, and subjective symptoms, specific questionnaires were administered to all enrolled workers [85]. Demands-control Model of the “Karasek” model is one of the most widely studied models of the two main work dimensions, the DL, referred to as the evaluation of skill and authority decision; and Psychological Demands (PD), referred to as workload and mental condition of workers [86,87]. These evaluations were resumed in the Italian version of JCQ to determine occupational stress. This test includes 46 items divided into 9 questions for DL, 20 for JD, 8 for SS, and 9 for JI. The analysis of the JD and DL score define the job strain variable as high JD and low DL [88]. Symptoms perceived by the workers like headache, nausea, and stomach-pain were collected during the medical examination. Moreover, we administered the STAI test to participants, in scale “1” to measure state-anxiety as a temporary and varying condition (STAI X-1), and in scale “2” to monitor trait-anxiety, as a relatively fixed personality tendency (STAI X-2) [89].

### 4.4. Saliva Sampling and Cortisol Analysis

Whole salivary samples of all enrolled workers were collected at 8:00 a.m. within 1 hour of waking up, in sterile 5 mL plastic tubes. To prevent sample contamination, participants were not allowed to smoke, eat or drink 15 min before saliva collection. Immediately after collection, the sample was centrifuged at 5000× *g*/min for 5 min to collect the supernatants that were stored at −80 °C until processing. Salivary cortisol concentration was carried out using a high-sensitivity enzyme immunoassay ELISA kit (BioVendor, Brno, Czech Republic). The assay was performed according to the manufacturer’s directions and read at 450 nm using a microplate reader (GloMax-Promega, Milan, Italy). Analytical sensitivity (lower limit of detection) was 1 ng/mL, and the intra- and inter-assay CVs were respectively 8.0% and 8.7%, which are within the acceptable ranges (i.e., accuracy ≤15%; intra-assay CV ≤10%; inter-assay CV ≤15%). No cross-reaction was detected with DHEAS and Tetrahydrocortisone.

### 4.5. Blood Sampling

Samples were collected at the same time and within 1 hour of waking up to reduce bias associated with the circadian cycles (08:00 A.M. and 9.00 A.M.). Twenty milliliters of whole blood samples were collected in heparinized vacutainers from peripheral veins according to the routine puncture method, during routine control by occupational medicine. Plasma was collected by blood centrifugation at 2000× *g* for 15 min and frozen at −80 °C within 30 minutes until assayed. PBMCs had been isolated from the heparinized blood by Ficoll-Hypaque (Merck, Darmstadt, Germany) gradient separation and washed three times with Phosphate buffered saline-PBS (Merck, Darmstadt, Germany). The cells were suspended in RPMI 1640 medium containing 10% heat-inactivated fetal calf serum (Merck, Darmstadt, Germany), 10 mM HEPES, 100 units/mL penicillin, and 100 µg/mL streptomycin (hereafter referred to as a complete medium) and incubated at 37 °C in a humidified and 5% CO2 incubator, with or without PHA (20 µg/mL) or LPS (5 µg/mL) for 24 h. At the end of incubation, supernatants and cell pellets were harvested and stored at −80 °C for subsequent analyses.

### 4.6. Circulating and Released Cytokines Analysis

Concentrations of selected Th1 and Th2 cytokines (IL-10; IL-6; TNFα and IL-1β) were measured in duplicate, in a single batch, using enzyme immunoassay Quantikine kit (R&D System, Inc., Minneapolis, MN, USA) in plasma and PBMCs supernatant. The assay was performed according to the manufacturer’s directions and read at 450 nm using a microplate reader (GloMax-Promega, Milan, Italy). IL-10: range 3.16–1000 pg/mL; sensitivity 0.5 pg/mL; intra-assay precision 2.5% (CV) inter-assay 9.8%(CV). IL-6: 10−3160 pg/mL; sensitivity 1 pg/mL; intra-assay precision 2.5% (CV) inter-assay 3.8%(CV). IL-1β: 3.9−250 pg/mL; sensitivity ≤0.15 pg/mL; intra-assay precision 5.3% (CV) inter-assay 5.8%(CV). TNFα: 15.6−1000 pg/mL; sensitivity ≤1 pg/mL; intra-assay precision 5.5% (CV) inter-assay 7.5%(CV).

### 4.7. Cytokine Gene Expression

Total RNA was extracted from PBMC after the culture, using TRIzol reagent (Invitrogen, Life Technologies, Paisley, UK) according to the manufacturer’s protocol. The RNA concentration was determined by measuring the samples’ absorbance at λ260nm by NanoDrop 2000 UV-Vis Spectrophotometer (Thermo Scientific, Waltham, MA, USA) and its purity was assessed by the absorbance ratio λ260/280nm and λ260/230nm. For each sample, 1μg of RNA was reverse-transcribed into complementary DNA using the QuantiTect Reverse Transcription Kit (Qiagen, Venlo, Limburg, NL). Subsequently, Real-Time PCR was performed using the GoTaq^®^ qPCR Master Mix (Promega, Madison, USA), cDNA and specific primer pairs, to evaluate the gene expression of IL-1β (FW 5′-TGAGGATGACTTGTTCTTTGAAG-3′, RW 5′-GTGGTGGTCGGAGATTCG-3′), TNFα (FW 5′-CCTTCCTGATCGTGGCAG-3′, RW 5′-GCTTGAGGGTTTGCTACAAC-3′), IL-6 (5-FWGTACATCCTCGACGGCATC-3′, RW 5′-ACCTCAAACTCCAAAAGACCA-3′), IL-10 (FW 5′-GAGAACCAAGACCCAGACATC-3′, RW 5′-TCACTCATGGCTTTGTAGATGC-3′). The housekeeping gene 18s (FW 5′-CTTTGCCATCACTGCCATTAAG-3′, RW 5′-TCCATCCTTTACATCCTTCTGTC-3′) was used as a reference gene. All PCR reactions were performed in triplicates in the Mastercycler Eppendorf (Eppendorf, Hamburg, Germany) with the following conditions: initially, 2 min incubation at 95 °C followed by 40 cycles consisting of 30 s at 95 °C, then 60 °C for 1 min, and 30 s at 68 °C. The analysis of the melting curve was performed in the temperature range of 60 to 95 °C at the end of each run. Quantification of gene expression was calculated using the comparative threshold cycle (Ct) method, normalized to the 18 s and efficiency of the RT reaction (relative quantity, 2^−∆∆Ct^). The replicates were then averaged, and the fold difference was determined, considering the value in the “Control” group as 1. Mean ± SD intensities were calculated for all Real-Time PCR experiments.

### 4.8. Statistical Analysis

Quantitative variables are summarized as the mean value and either standard deviations (SD) or standard error (SEM) in the Tables and Figures. Qualitative variables are represented as frequency and percentage. For statistical analysis, quantitative data were analyzed by Student t-test for unpaired data or Mann-Whitney U test, according to their distribution evaluated by the Shapiro-Wilks test.

All tests were two-tailed and viewed as indicating statistical significance at a *p*-value ≤ 0.05. Analyses were performed by SPSS Inc. statistical software package (Version 23.0).

## Figures and Tables

**Table 1:** Clinical-demographic details of overall workers and in workers with cortisol levels ≤10 or >10 ng/mL.

Parameters	Total Workers	Cortisol ≤10 ng/mL	Cortisol >10 ng/mL	*p*-value
Number of workers	80	31	49	
Age (years)	36.8 ± 9.3	37.9 ± 9.6	35.8 ± 9.3	0.325
Weight (kg)	91.3 ± 12.3	84.0 ± 9.9	98.5 ± 14.7	<0.001
Height (cm)	176.3 ± 19.9	180.3 ± 6.4	172.3 ± 33.5	0.191
BMI (kg/m^2^)	27.2 ± 2.9	26.0 ± 2.5	28.5 ± 3.4	<0.001
Systolic blood pressure (mmHg)	124.3 ± 8.8	125.2 ± 11.7	123.4 ± 12.2	0.517
Diastolic blood pressure (mmHg)	72.8± 11.5	72.5 ± 11.6	73.0 ±11.3	0.841
STAI X-1	44.1 ± 4.4	43.5 ± 3.2	44.7 ± 5.7	0.305
STAI X-2	38.6 ± 5.3	37.6 ± 5.2	39.5 ± 5.1	0.100
Job Demand (JD)	34.2 ± 4.3	30.9 ± 4.6	37.6 ± 3.9	<0.001
Decision Latitude (DL)	64.5 ± 6.4	62.83 ± 5.6	66.2 ± 7.2	0.027
JD/DL (Job Strain)	0.53 ± 0.07	0.49 ± 0.08	0.56 ± 0.07	<0.001
Social Support (SS)	22.1 ±2.8	22.8 ± 3.0	21.4 ± 2.7	0.041
Job Insecurity (JI)	22.1 ±4.8	23.0 ± 5.0	21.1 ± 4.6	0.084

**Table 2:** Number of blood cells in overall workers and workers with cortisol levels ≤ or >10 ng/mL.

Parameters	Total Workers	Cortisol ≤10 ng/mL	Cortisol >10 ng/mL	*p-*value
WBC (10^3^/µL)	6.75 ± 1.82	6.56 ± 1.92	6.94 ± 1.72	0.739
RBC (10^6^/µL)	5.01 ±0.56	4.96 ± 0.33	5.07 ± 0.23	0.677
HGB (g/dL^−1^)	15.39 ±1.70	15.34 ± 0.82	15.45 ± 0.88	0.868
Platelets (10^3^/µL)	218.32 ± 36.68	223.1 ± 46.05	213.54 ± 27.31	0.054
% limphocytes	36.14 ± 7.69	36.83 ± 8.31	35.41 ± 7.08	0.803
% monocytes	8.94 ± 1.73	8.96 ± 1.94	8.92 ± 1.53	0.967
% neutrophils	53.28 ± 8.41	52.91 ± 9.31	53.64 ± 7.52	0.868
% eosinophils	1.75 ± 0.85	1.61 ± 0.62	1.94 ± 1.08	0.008
% basophils	0.06 ± 0.04	0.06 ± 0.04	0.06 ± 0.04	1.000

**Table 3:** Characteristics of enrolled gas and oilfield workers.

Behavior Data	
Current smoker, *n* (*%*)	15 (18.7%)
Cigarettes/d range	3–5
Alcohol consumption, *n* (*%*)	25 (31.25%)
Wine or beer glasses/d	One or twice a day
Complaints	
Diarrhea, *n* (*%*)	6 (7.55 %)
Abdominal pain, *n* (*%*)	5 (6.25%)
Headaches and dizziness, *n* (*%*)	4 (5 %)
Cold, *n* (*%*)	2 (2.5 %)
Educational grade	
Secondary school, *n* (*%*)	65 (81.25%)
high school, *n* (*%*)	15 (18.75%)
Occupational grade	
Coordinator, *n* (*%*)	9 (11.25%)
Supervisor, *n* (*%*)	12 (15%)
Operator, *n* (*%*)	59 (73.75%)
Marital status	
Single, *n* (*%*)	30 (37.5%)
Married, *n* (*%*)	50 (62.5%)

## Abbreviations

CNS	Central Nervous System
Th	T helper lymphocytes
IFN	Interferon
IL	Interleukin
TNF	Tumor Necrosis Factor
HPA	Hypothalamic–Pituitary–Adrenal
STAI	State-trait-anxiety inventory
JD	Job Demand
DL	Decision Latitude
SS	Social Support
JI	Job Insecurity
BMI	Body Max Index
PBMCs	Peripheral Blood Mononuclear Cells
PHA	Phytohaemagglutinin
LPS	Lipopolysaccharide
JCQ	Job Content Questionnaire
NF-κB	nuclear transcription factor- κB
AD	Alzheimer’s disease
PD	Psychological Demands
